# Supplementary material for: Food odors trigger Drosophila males to deposit a pheromone that guides aggregation and female oviposition decisions
Source: eLife. 2015 Sep 30;4:e08688. doi: 10.7554/eLife.08688 (PMC4621432; doi:10.7554/eLife.08688)
Supplement: Supplementary file 1. — Volatile male-specific and male-enriched pheromones detected by GC–MS under control and experimental conditions. DOI: http://dx.doi.org/10.7554/eLife.08688.049 [file elife08688s008.docx]

**Supplementary File 1. Volatile male-specific and male-enriched pheromones detected by GC-MS under control and experimental conditions. *^a^***

| **Volatile Pheromone** | **ACV-only** | **HA+flies** | **ACV+flies** | **Fold Enriched** |
| --- | --- | --- | --- | --- |
| cis-vaccenyl acetate (cVA) | N.D. ***^b^*** | N.D. | N.D. | **-** |
| 7-docosene (7-D) | N.D. | N.D. | N.D. | **-** |
| 5-tricosene (5-T) | N.D. | trace ***^c^*** | trace | **-** |
| 9-tricosene (9-T) | N.D. | 15.2 ± 3 .2 | 42.2 ± 14 | **2.8** |
| 2-methyldocosane (23-Br) | N.D. | N.D. | N.D. | **-** |
| 7-tricosene (7-T) ***^d^*** | N.D. | 158.6 ± 36 | 552.9 ± 225 | **3.5** |

***^a^* values normalized by internal standard (hexacosane n-C26)**

***^b^* N.D., not detected;**

***^c^* trace, not detected on TIC; detected only by characteristic ion (*m/z* 97)**

***^d^* male-enriched but not specific**
